# Social Capital and Family Well-Being Among Immigrant Chinese and Native Japanese Families Raising Children in Japan: A Cross-Sectional Study

**DOI:** 10.3390/healthcare13131518

**Published:** 2025-06-25

**Authors:** Qiting Lin, Takafumi Soejima, Shiqi Zhang, Hisashi Nakaguchi, Satoshi Takatani, Junko Honda, Naohiro Hohashi, Noriyuki Nishimura

**Affiliations:** 1 Graduate School of Health Sciences, Kobe University, Kobe 654-0142, Japan; fumi.soeji@gmail.com (T.S.); 222k053k@stu.kobe-u.ac.jp (S.Z.); 165k006k@stu.kobe-u.ac.jp (H.N.); takatani@otemae.ac.jp (S.T.); naohiro@hohashi.org (N.H.); nnishi@med.kobe-u.ac.jp (N.N.); 2 Research Institute of Nursing Care for People and Community, University of Hyogo, Akashi 673-8588, Japan; junko_honda@cnas.u-hyogo.ac.jp

**Keywords:** social capital, family well-being, cross-sectional observational study, family raising children in Japan

## Abstract

**Background/Objectives**: Although several studies have examined the importance of social capital to individual health, the relationship between social capital and family well-being remains unclear. This study examines the relationships among social capital, parental depressive symptoms, and family well-being, and evaluates whether parental depressive symptoms mediate the association between social capital and family well-being in immigrant Chinese and native Japanese families. **Methods**: A cross-sectional study using Google questionnaires was conducted between January and April 2024 among parents of local Japanese and immigrant Chinese families with children. The survey assessed demographics and social capital using the PSCS-16 and K6 Scale, family well-being using the General Functioning Index of the Family Assessment Device, and family life satisfaction using the New Brief Job Stress Questionnaire. Multiple-group structural equation modeling clarified the association between bonding and bridging social capital and family well-being. **Results**: The survey data was collected from 133 parents (75 Japanese and 58 Chinese) of preschool children aged six years and under. About 81.2% of respondents were mothers and 18.8% were fathers, with the majority aged between 30 to 39 years (63.9%). Bonding social capital was positively associated with family well-being among Japanese families. In Chinese families, bonding social capital was indirectly associated with family well-being by reducing parental depressive symptoms. **Conclusions**: These findings highlight the importance of enhancing bonding social capital to benefit both native and immigrant families. Besides, policymakers should consider tailored strategies that reflect the differing needs of both groups.

## 1. Introduction

The population of foreign nationals residing in Japan reached 3.22 million in 2023, accounting for 2.59% of Japan’s total population [1]. Among them, Chinese nationals constituted the largest proportion (24.8%) [1]. Most Chinese foreign nationals were permanent residents (314,354 people), and long-term residents and family-stay individuals comprised 94,685 [1]. The number of married Chinese people raising children in Japan is likely to increase in the future. The Japanese Ministry of Internal Affairs and Communications formulated the Plan for the Promotion of Multicultural Coexistence to assist immigrant families with adjusting to Japanese society by providing social support and a conducive parenting environment [2]. Despite increasing attention on policy, little is known about how Chinese immigrant families navigate and mobilize resources supporting their well-being.

Social capital refers to the resources accessible through social networks and relationships built on trust and reciprocity [3,4]. Putnam [3] defines social capital as individuals’ connections, especially social networks, that foster mutual trust and cooperation. The two primary types are bonding social capital, which connects individuals with close ties such as family and friends, and bridging social capital, which links individuals to broader social groups such as political or economic organizations [3,5,6,7]. Previous research has examined the link between various forms of social capital (cognitive, structural, bonding, bridging) and well-being [3,4,5,6,7,8,9,10,11,12]. A meta-analysis found that these social capital types relate to mental health, mortality, and disease outcomes [8]. Elements such as social support, trust, and civic engagement also correlate with self-reported health [9]. Bonding social capital typically involves close-knit relationships that provide emotional and practical support, while bridging social capital offers access to information and services through wider social networks, contributing to health benefits [5,6,8,10,11,12]. For example, bonding ties assist individuals in coping with life changes, particularly among older adults, whereas bridging ties broaden resources and opportunities [5]. While social capital benefits families in general, immigrant families may face unique challenges in building and maintaining such resources. For example, previous studies have indicated that Chinese parents raising children in Japan experience several challenges such as high parenting stress, lack of social support, and poor mental and physical health [9,13,14]. Understanding how social capital functions in this context may offer valuable insights into how immigrant families navigate parenting-related challenges and sustain family well-being in the host society.

Bonding and bridging social capital have been widely studied among immigrant populations and shown to positively influence immigrants’ health, including their psychological well-being and emotional expression [5,7,15,16,17]. For Chinese residents in Japan, bonding social capital, such as emotional and financial support, and bridging social capital, such as access to job information and social companionship, are important factors affecting their psychological distress [17]. Research from the United States indicated that bonding social capital, including financial and parenting support, is a stronger predictor of maternal well-being for immigrant Latina mothers than for native Latina mothers [7]. Similar associations between social capital and emotional expression have been observed among Portuguese immigrant and native populations [15]. Social capital may contribute to both individual and family well-being. For example, Japanese families raising children in the US faced challenges in utilizing resources from Japan and building connections with local communities, including Japanese networks, which led to decreased family functioning and life satisfaction [18]. Strong internal family bonds, such as those between parents and children, help foster belonging, emotional support, and family cohesion [19], enabling families to better access external resources and improve overall functioning [19]. Consequently, social capital supports mental health and enhances family well-being. Despite these insights, the role of bonding and bridging social capital in promoting the well-being of immigrant families remains underexplored.

Family Systems Theory states that an individual family member’s health and the family’s overall health mutually influence each other [20]. Any change in a family member’s physical and psychological well-being affects the entire family’s dynamics and functioning [21]. According to Family Systems Theory [21] and previous research [15,16,17,18,19], social capital influences family well-being through two pathways: (1) a direct relationship between social capital and family well-being and (2) an indirect relationship mediated by the psychological health of family members. Investigating both direct and indirect links between social capital and family well-being can deepen our understanding of their connection. Such insights are valuable for developing policies and community programs that strengthen family support, especially for immigrant families raising children, ultimately enhancing their overall well-being. Although social capital has been shown to promote health and well-being among immigrant groups, it remains unclear whether social capital’s role differs between immigrant and native families living in the same sociocultural context within an Asian society.

Comparing immigrant Chinese families with native Japanese families can help reveal whether the mechanisms through which social capital contributes to family well-being differ across cultural or contextual lines within an Asian society. Given that Chinese immigrant families in Japan often face language barriers and have limited familiarity with local systems and resources [9,13], they may require a greater level of bonding social capital to navigate child-rearing and maintain family well-being.

## 2. Materials and Methods

### 2.1. Hypotheses and Conceptual Framework

This study examines the relationship between bonding social capital and bridging social capital and family well-being, which includes both family functioning and family life satisfaction. The analysis focuses on two distinct population groups, namely immigrant Chinese families and native Japanese families who are raising children in Japan. By comparing these two cultural groups within the same national setting, this study seeks to understand whether the pathways connecting social capital, psychological well-being, and family well-being differ based on cultural background.

We formulated the following hypotheses:

**H1.**  *Bonding social capital and bridging social capital are directly associated with the family well-being of both immigrant Chinese families and native Japanese families.*

**H2.**  *The relationship between bonding social capital and bridging social capital and family well-being is mediated by parental depressive symptoms in both Chinese and Japanese families.*

**Figure 1:**
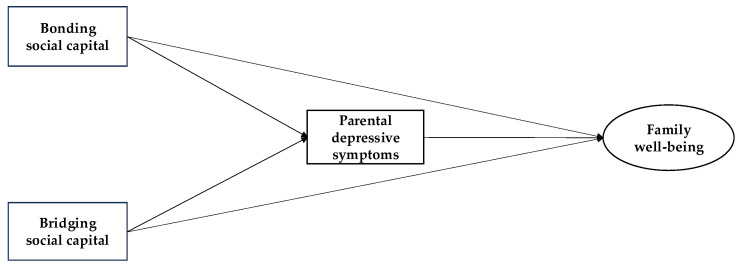
Theoretical framework of the relationships between bonding social capital, bridging social capital, parental depressive symptoms, and family well-being.

**H3.**  *Bonding social capital has a stronger association with family well-being among Chinese families than among Japanese families (See Figure 1)*.

### 2.2. Research Design, Participants, Setting, and Period

A cross-sectional survey was conducted from January to April 2024 using self-administered questionnaires via Google Forms. The participants included immigrant Chinese and native Japanese families raising preschool-aged children in Japan. For Japanese families, eligibility criteria were: (1) both parents held Japanese nationality; (2) the youngest child was between 0 and 6 years old and living in Japan; and (3) parents comprehended the study objectives and completed the questionnaire in either Japanese or Chinese. For Chinese families, the criteria were similar: (1) both parents were Chinese nationals; (2) the youngest child was a preschooler (0–6 years) living in Japan; and (3) parents understood the study purpose and answered the questionnaire in Japanese or Chinese.

### 2.3. Procedure

First, convenience sampling was employed to recruit participants from four child-related institutions located in an urban area of Japan, including two nursery schools, a children’s center, and an international school. The study’s purpose was explained either in written form or orally to administrators at each facility, and formal approval for participation was obtained from all institutions involved. Second, in the nursery schools and children’s center, staff distributed printed flyers containing QR codes to parents. These QR codes were linked to an online survey hosted on Google Forms, which included study details and the informed consent form. At the international school, a PDF version of this flyer was distributed by school staff via email. Third, participant recruitment among Chinese families was supplemented by implementing a snowball sampling method. Researchers sent the PDF flyer to Chinese-speaking communities and parent groups on social platforms in Japan, such as WeChat, including those related to shopping, parenting, or secondhand goods. Eligible parents were also contacted directly by researchers or through personal networks. Fourth, individuals interested in participating accessed the survey using the QR code provided. After reading the study description, participants confirmed their willingness to participate by selecting an electronic consent option (“I agree”). If individuals did not wish to participate, they were advised to close the webpage.

### 2.4. Measures

#### 2.4.1. Demographics

The demographic data collected from participants included nationality (Japanese or Chinese), parental role (father or mother), age of participant and spouse, length of residence in Japan, education level, marital status, employment status, household composition, economic status, number of children, age and sex of the youngest child, and whether any children had chronic illnesses or disabilities.

#### 2.4.2. Social Capital

The Personal Social Capital Scale (PSCS) [4,10,11] was employed to assess participants’ social capital. This instrument contains 16 items divided into two subscales: bonding social capital (items 1 to 8) and bridging social capital (items 9 to 16). Each item is rated on two separate five-point Likert scales. For the “network size” dimension, response options were a few (1), less than average (2), average (3), more than average (4), and a lot (5). For the “number of network members” dimension, choices included none (1), a few (2), some (3), most (4), and all (5). Scores for each subscale were calculated by summing the responses to their respective items, with total possible scores ranging from 8 to 40. Higher scores indicate greater bonding or bridging social capital.

The Chinese adaptation of the PSCS exhibited good reliability and validity [4]. In our sample, the Chinese PSCS demonstrated a Cronbach’s alpha of 0.885. The Japanese version underwent forward-backward translation, and its linguistic and cultural equivalence was confirmed by two researchers who are bilingual in Chinese and Japanese. In our sample, the Japanese version of the PSCS showed a Cronbach’s alpha of 0.844. Additionally, multitrait item correlation analysis confirmed the factorial validity of the Japanese version, showing scaling success rates of 87.5% (bonding subscale) and 100% (bridging subscale) [22].

#### 2.4.3. Depressive Symptoms

The Kessler Psychological Distress Scale (K6) was utilized to assess depressive symptoms in parents [23]. The scale, available in both Japanese and Chinese, consists of six items rated on a five-point Likert scale: none of the time (0), a little of the time (1), some of the time (2), most of the time (3), and all of the time (4). Scores from all items were summed to generate a total score ranging from 0 to 24, where higher scores indicate greater psychological distress [23,24,25]. Previous studies have confirmed good reliability and validity for both language versions [24,25]. In the present sample, Cronbach’s alpha values were 0.843 and 0.862 for the Japanese and Chinese versions, respectively.

#### 2.4.4. Family Well-Being

The General Functioning Index (GFI) from the Family Assessment Device (FAD) [26] was employed to evaluate family functioning among Japanese and Chinese families raising children in Japan. Both language versions of the measure have demonstrated strong reliability and validity in prior studies [27,28]. The GFI consists of 12 items rated on a four-point Likert scale: strongly agree (1), agree (2), disagree (3), and strongly disagree (4). Half of these items are positively phrased (e.g., “During a crisis, we can turn to each other for support”), while the other half are negatively phrased (e.g., “We cannot talk to each other about the sadness we feel”), with the positive items reverse scored so that higher total scores reflect worse family functioning. The total GFI score ranges from 12 to 48 points. In the present study, Cronbach’s alpha was 0.842 for the Japanese version and 0.910 for the Chinese version. Additionally, family life satisfaction was measured by the item “I am satisfied with my family life” from the New Brief Job Stress Questionnaire [29]. Respondents selected from options ranging from very unsatisfactory (1), unsatisfactory (2), dissatisfied (3), and satisfied (4), where higher scores indicate greater satisfaction. The reliability and validity of this questionnaire have been confirmed in samples of 1633 Japanese workers [29] and 568 Chinese workers [30].

### 2.5. Statistical Analysis

First, descriptive statistics including frequencies, percentages, means, and standard deviations (SD) were computed for demographic characteristics, social capital, depressive symptoms, family functioning, and family life satisfaction. Comparisons between Japanese and Chinese family participants were conducted using independent samples *t*-tests or Mann–Whitney U tests for continuous variables, and chi-square tests for categorical variables. To investigate potential confounders, bivariate relationships among family functioning, family satisfaction, social capital, depressive symptoms, and demographic factors were analyzed using Spearman’s rank correlation.

Subsequently, multiple-group structural equation modeling (SEM) was performed to examine the relationships among bonding and bridging social capital, parental depressive symptoms, family functioning, and family satisfaction in both Chinese and Japanese families raising children. SEM was selected because it allows for the simultaneous estimation of direct and indirect associations among social capital, parental depressive symptoms, and family well-being, and multiple-group SEM enables the comparison of structural relationships across immigrant Chinese and native Japanese families. Demographic factors showing significant correlations with key variables in the bivariate analysis (such as economic status) were incorporated into the model to evaluate their influence on model fit. Multiple models were compared: (1) models with and without selected demographic variables; (2) models with path coefficients constrained to be equal across Japanese and Chinese groups versus the unconstrained model. Specifically, seven models were tested: Model 1 (unconstrained), Model 2 (all paths constrained), and Models 3–7, where individual path constraints were applied to assess group differences in specific pathways.

Model fit was assessed using multiple indices, including the chi-square statistic relative to degrees of freedom (CMIN/df), comparative fit index (CFI), adjusted goodness-of-fit index (AGFI), goodness-of-fit index (GFI), root mean square error of approximation (RMSEA), and Akaike’s information criterion (AIC) [31]. Criteria for a good fit were defined as CMIN/df less than 2, CFI greater than 0.90, AGFI above 0.95, GFI exceeding 0.90, and RMSEA below 0.05 [31]. Data analyses were performed with IBM SPSS Statistics version 29.0 and AMOS version 29.0 (IBM Corp., Armonk, NY, USA). Statistical significance was set at *p* < 0.05 (two-tailed).

### 2.6. Ethical Considerations

The Ethics Review Committee of the lead researcher’s institution approved this study (approval number: 1219).

## 3. Results

### 3.1. Participant Recruitment

Approximately 860 flyers were handed out to potential participants: 360 at two nursery schools, 500 at one international school, and 50 at one children’s center. In addition, a PDF version of the flyer was shared with more than 2000 potential participants. Using convenience and snowball sampling methods, a total of 140 parents were recruited during the survey period. Due to the combined use of flyer distribution and online snowball sampling, it was not feasible to calculate an exact response rate. Among the recruited parents, one respondent reported a nationality other than Japanese or Chinese, one respondent was single and their youngest child was older than six, three respondents were Chinese nationals married to Japanese spouses, and two respondents were Chinese nationals with spouses of other nationalities. Ultimately, valid data from 133 respondents with preschool-aged children (0–6 years old) were included in the analysis.

### 3.2. Participant Characteristics

Data from 133 parents were included in the analysis, with 75 (56.4%) from Japanese families and 58 (43.6%) from Chinese families (see Table 1). Among the participants, 108 (81.2%) were mothers. The majority of participants (85, 63.9%) and their spouses (75, 56.4%) were aged between 30 and 39 years. Most participants (107, 80.5%) had completed university or graduate education. The average age of the youngest child was 2.74 years (SD = 2.12). Nearly half of the children were girls (65, 48.9%) and firstborns (64, 48.1%). Six children (4.5%) had chronic illnesses or disabilities.

### 3.3. Bivariate Analyses

Compared to Chinese families, participants from Japanese families were more often mothers (χ^2^ = 10.09, *p* = 0.001) and more frequently lived in a nuclear family structure (χ^2^ = 4.135, *p* = 0.042) (see Table 1). Japanese participants also reported a higher perceived economic status than their Chinese counterparts (χ^2^ = 20.63, *p* < 0.001). By contrast, Chinese family participants exhibited greater levels of depressive symptoms (Z = 2.29, *p* = 0.004), poorer family functioning (Z = −3.23, *p* = 0.001), and lower satisfaction with family life (Z = 3.10, *p* = 0.001).

In Spearman’s rank correlation analysis, the bonding social capital score was significantly correlated with family functioning (*r* = −0.27, *p* = 0.002) and family satisfaction (*r* = 0.26, *p* = 0.003), although bridging social capital was not. Participants’ depressive symptoms were correlated with family functioning (*r* = 0.45, *p* < 0.001) and family satisfaction (*r* = −0.25, *p* = 0.003) (Table 2). Additionally, economic status was significantly and negatively correlated with depressive symptoms (*r* = −0.177, *p* = 0.041), and female participants (mothers) reported lower family functioning than did male participants (fathers) (*r* = −0.24, *p* = 0.006). Therefore, both economic status and gender were included in the structural model.

### 3.4. Multiple-Group SEM Among Japanese and Chinese Families

The original multiple-group SEM model included the main variables and a demographic variable that was significantly correlated with the main variables in the bivariate analyses as adjusted variables (i.e., participants’ relationship with children and economic status). Compared to the adjusted model (CMIN/df = 0.694, CFI = 1.000, AGFI = 0.925, GFI = 0.969, RMSEA = 0.000, AIC = 81.953), the model with only the primary variables demonstrated a better fit (CMIN/df = 0.513, CFI = 1.000, AGFI = 0.947, GFI = 0.984, RMSEA = 0.000, AIC = 64.667). Therefore, we selected the model with only the primary variables.

In comparing the models with and without path constraints, the model that constrained the path from bridging social capital to family well-being (Model 6) demonstrated a better fit (CMIN/df = 0.209, CFI = 1.000, AGFI = 0.981, GFI = 0.997, RMSEA = 0.000, AIC = 51.044) (Table 3). Therefore, we used Model 6 for further analysis.

**Figure 2:**
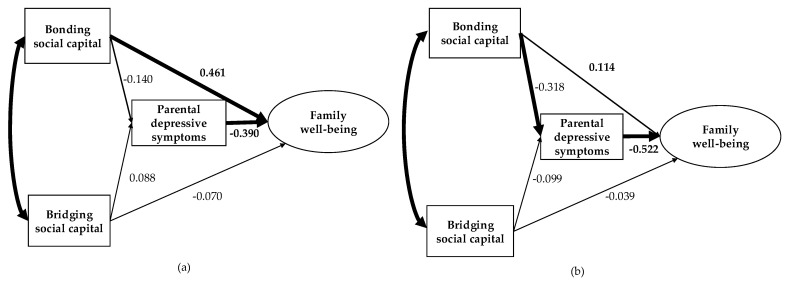
Multiple-group analysis in structural equation modeling: (**a**) Japanese families (*n* = 75) and (**b**) Chinese families in Japan (*n* = 58). Note. Bonding social capital and bridging social capital were measured by the Personal Social Capital Scale 16; parental depressive symptoms were measured by the Kessler Psychological Distress Scale (K6); and family well-being was modeled as a latent variable composed of two observed indicators: family functioning was measured by the General Functioning Index of the Family Assessment Device and family satisfaction was measured by the New Brief Job Stress Questionnaire. Significant paths are highlighted in bold. Although unstandardized path coefficients from bridging social capital to family well-being in the model between Japanese and Chinese families were constrained to be equal, the standardized path coefficients are shown separately for each group. In the path diagram, bold arrows indicate statistically significant paths (*p* < 0.05), whereas thin arrows indicate non-significant paths. Standardized path coefficients are displayed next to each arrow. Measurement errors for the observed variables (e1–e4) were included in the model but omitted from the figure for clarity.

The final model revealed that, among participants from Japanese families, bonding social capital directly enhanced family well-being as a latent variable of family functioning and family life satisfaction (β = 0.461; 95% confidence interval 0.213–0.584; *p* < 0.001), while parental depressive symptoms directly diminished family well-being (β = −0.390; 95% confidence interval −0.692–−0.063; *p* = 0.002) (Figure 2). Furthermore, among participants from Chinese families, bonding social capital reduced parental depressive symptoms (β = −0.318; 95% confidence interval −0.390–−0.060; *p* = 0.016), and parental depressive symptoms affected family well-being (β = −0.522; 95% confidence interval −1.189–−0.482; *p* < 0.001). This suggests that bonding social capital positively influences family well-being through parental depressive symptoms. A comparison of the impact of bonding social capital on family well-being indicated that its total effect was greater in Japanese families (0.516; 95% confidence interval 0.243–0.714; *p* = 0.001) than in Chinese families (0.280; 95% confidence interval 0.047–0.521; *p* = 0.037).

## 4. Discussion

The study results indicated that bonding social capital was significantly associated with family well-being in Japanese families and indirectly associated with family well-being in Chinese families in Japan, confirming parts of Hypotheses 1 and 2. However, contrary to Hypothesis 3, bonding social capital shows a stronger association with family well-being in Japanese families than in Chinese immigrant families. Furthermore, structural equation modeling (SEM) revealed that parental depressive symptoms showed statistically meaningful relationships with family well-being in both Chinese and Japanese families. In particular, greater depressive symptom severity in parents corresponded to lower levels of family functioning and satisfaction. This finding supports Family Systems Theory [20,21], which emphasizes that individual psychological well-being and overall family functioning are dynamically interrelated. When a parent experiences psychological distress, it may impair communication and overall relationship quality within the family, thereby diminishing overall family well-being. These results underscore the mutual influence between individual and family-level health outcomes within the Family Systems Theory framework [20,21]. This study highlights the distinct pathways through which social capital may be related to the well-being of both local Japanese and immigrant Chinese families. These differences suggest that culturally and contextually tailored strategies may be more appropriate for addressing the needs of immigrant Chinese families. Nevertheless, as the comparison of model fit across constrained and unconstrained models remains inconclusive, it would be premature to assert that social capital has culturally distinct effects on family well-being.

Our findings indicate that Chinese participants tended to report experiencing more depressive symptoms than did their Japanese counterparts. This aligns with previous research suggesting that Chinese immigrant mothers in Japan often face mental health challenges, potentially due to the stress of parenting and cultural adjustment [4]. By contrast, a previous study found that both married and single Chinese immigrants in Japan generally reported experiencing lower levels of depressive symptoms [17], indicating that the psychological distress observed in our sample may be more characteristic of parents raising children. This observation is consistent with earlier studies on Japanese immigrant families in the U.S., where limited access to both internal and external support systems normally available in their home country was associated with reduced family functioning [18]. For Chinese families raising preschool-aged children in Japan, similar challenges may arise due to limited availability of familial and community-based support networks [9,13]. Moreover, linguistic difficulties and the possibility of social isolation may intensify family stress, affecting the quality of family life [32,33].

Notably, this study found that bonding social capital was indirectly associated with family well-being among Chinese families, via its association with lower levels of parental depressive symptoms. One possible explanation for this indirect association is that receiving emotional support from relatives and friends may help mitigate psychological distress among Chinese parents, which in turn is associated with improved perceptions of family well-being. In an extended family structure, relatives often provide both emotional and instrumental support, as highlighted in previous research [9,13]. In Chinese cultural contexts, caregiving responsibilities are often shared with mothers and mothers-in-law, who commonly help care for infants and reduce the parenting burden on the younger generation [13,34]. Emotional support has also been reported to contribute to the psychological well-being of Chinese immigrants living in Japan [17,35]. Our data revealed that 86.2% of the study participants lived in a nuclear family structure consisting of parents and their children. For many Chinese parents in Japan, it can be difficult to obtain practical help from extended family members or friends during emergencies, such as when both parents are unavailable to care for a sick child due to work obligations. Geographic separation often limits the availability of hands-on childcare support, although emotional support may still be accessible. In this context, bonding social capital, mainly reflected in emotional support from close connections, may be associated with reduced psychological distress and better perceptions of family well-being.

By contrast, bonding social capital in Japanese families was directly associated with family well-being, and no indirect link with parental depressive symptoms was observed in the model. The Japanese government established the Children and Families Agency to provide support, such as psychological counseling and child abuse prevention, to families during the child-rearing period [36]. These public services offer parents relatively easy access to professional mental health support. Despite the availability of these services, a qualitative study found that Japanese parents often attempt to manage psychological distress on their own or delay seeking help from professionals, rather than turning to extended family and friends for support [37]. Such help-seeking behaviors may weaken the potential mediating role of bonding social capital in improving mental health. Conversely, Chinese parents experiencing mental health issues might face challenges in accessing professional counseling due to language barriers [18,38]. This could be one factor contributing to the connection observed between bonding social capital and family well-being via parental depressive symptoms specifically in Chinese parents.

This study shows that family well-being has a greater association with bonding social capital among Japanese families with children than among Chinese families with children in Japan. This variation could be attributed to the participants’ social backgrounds. Within families, social capital encompasses the collective assets accessible through members’ social networks [6]. These assets can shape family well-being by influencing the dominant social norms, values, and attitudes shared within these connections. In many Asian societies, the cultural importance placed on strong family bonds and community support likely amplifies the beneficial effects of bonding social capital [39]. In addition, compared with Chinese families living in Japan, native Japanese families may possess more established and effective bonding networks, as they are more likely to have long-standing connections with relatives, neighbors, and local communities. Nevertheless, Chinese families residing in Japan might encounter distinct social dynamics and support structures [9,13], which could affect the role of bonding social capital and, consequently, family well-being. These families often face difficulties, such as cultural adaptation, language obstacles, and a lack of sufficient practical support from relatives and friends, potentially diminishing the positive effects of bonding social capital on family well-being [9,13,15]. Thus, it is important to assess the impact of bonding social capital on family well-being within diverse social environments.

Statistically, no notable association was found between bridging social capital and parental depressive symptoms or family well-being among both Japanese and Chinese families. This result could be attributed to the characteristics of bridging social capital, which connects different social groups and provides access to resources and information [7,8,10,11]. In East Asian cultures, the concept of interdependent self-construal plays an important role in shaping social relationships [40]. Specifically, interdependence is salient in relationships involving kin and familiar others, whereas interactions with more distant or unfamiliar groups, such as institutions or broader social networks, are often characterized by an independent stance [40,41,42]. For instance, a multilevel study in Japan found that bridging social capital at the neighborhood level, which reflects weak ties, was not associated with lower levels of depressive symptoms among older adults [43]. Accordingly, in both Japanese and Chinese contexts, familiar groups are generally perceived as more trustworthy and supportive, while relatively unfamiliar groups may not provide the same level of emotional connection or perceived reliability. Although bridging social capital often provides important access to new opportunities and supports social inclusion [44], the cultural norms and values within Japanese and Chinese families may shape how bridging social capital is perceived and utilized. This cultural influence could restrict its direct impact on individual well-being as well as its role in improving family well-being [45]. Moreover, these non-significant findings may partly reflect measurement limitations, as the PSCS-16 scale might not fully capture immigrant-specific bridging ties, such as informal ethnic networks or culturally embedded supports.

These findings emphasize the connection between bonding social capital and family well-being in Japanese and Chinese families raising children. To improve the welfare of immigrant families with children, Japan introduced the updated 2020 Plan for the Promotion of Multicultural Coexistence to offer additional measures focused on child-rearing and welfare services. In accordance with this plan, current initiatives supporting immigrant families involve deploying bilingual staff to address language barriers and offering educational programs tailored for families from multicultural backgrounds [46]. Moreover, reinforcing psychological support and social networks for immigrant families raising children is essential. For instance, developing support groups for parents with similar cultural backgrounds could strengthen social ties among immigrant parents. Governments and local organizations could establish mental health support programs that include multilingual counseling services and peer support networks for immigrant parents. Finally, providing cultural training to healthcare providers would facilitate a better understanding of the cultural values and parenting norms of immigrant families, thus enhancing the accessibility and quality of support.

Our study contributes novel insights by shifting the focus from individual-level to family-level outcomes, thereby broadening the understanding of how social capital functions within nuclear families across cultural contexts. Despite its strengths, this study has several limitations. First, the generalizability of the findings may be limited due to the relatively small sample size, particularly the small proportion of Chinese families with children. The sampling strategy involved distributing questionnaires at nursery schools, children’s centers, and an international school, supplemented by snowball sampling via the researchers’ networks and participant referrals, mainly conducted through WeChat. This approach may have introduced sampling bias by favoring families with stronger existing social networks. Consequently, the findings may not be fully applicable to immigrant families with weaker social ties or fewer available support resources. Moreover, the small sample size may have reduced the statistical power to detect small or moderate associations, particularly within the Chinese group, possibly contributing to the lack of significant findings in some analyses. Second, the participants might possess a higher social status than that of the general Japanese population, as 80.5% had completed a university education and 77.5% reported an above average economic status. Notably, Japanese families reported significantly higher perceived economic status than Chinese immigrant families, which may have influenced social capital and family well-being. Families with elevated educational attainment and economic resources are more likely to have better access to social support networks, resources, and healthcare services [47,48]. Therefore, the findings may not be generalizable to families with lower socioeconomic status. This economic inequality between groups should be acknowledged as a limitation of the study and warrants further investigation in future research. Third, because the study uses a cross-sectional design, it cannot establish causal links between social capital, parental depressive symptoms, and family well-being. Therefore, the directionality and underlying mechanisms of these associations remain unclear. Fourth, our study did not identify whether social capital originated from ties within the same ethnic community (e.g., Chinese) or those from the broader host society, which limits the understanding of how different types of social capital influence immigrant family well-being. Future studies should adopt a longitudinal design to better understand the directionality and underlying mechanisms of the observed associations. It is also important to explore how these pathways differ across socioeconomic strata, particularly among disadvantaged families. In addition, future work should investigate the role of ethnic composition in social networks, and how it shapes both the accessibility and function of bonding and bridging social capital within immigrant populations.

## 5. Conclusions

This cross-sectional study examined the associations between bonding and bridging social capital and the relationship between parental depressive symptoms and family well-being, based on data from immigrant Chinese and native Japanese families raising children in Japan. The findings provide valuable empirical insight into how social capital’s role in family well-being may differ by cultural context. Moreover, implementing culturally tailored interventions to strengthen social capital and enhance mental health support could more effectively contribute to a higher level of family well-being. While the association between bonding social capital and family well-being was different between Japanese families and Chinese families, the causal relationships could not be established due to the study design. To advance this line of research, future studies should incorporate longitudinal designs and include culturally and socioeconomically diverse populations, in order to uncover causal relationships and better account for contextual and cultural variability.

## Figures and Tables

**Table 1:** Demographic characteristics of participants (*n* = 133).

	Total *n* (%)	Japanese *n* (%)	Chinese *n* (%)	χ^2^	*p* ^a^
Participants’ relationship with Children				10.09	0.001
Mother	108 (81.2%)	68 (90.7%)	40 (69%)		
Father	25 (18.8%)	7 (9.3%)	18 (31%)		
Participants’ age				5.48	0.065
20–29	16 (12%)	9 (12.0%)	8 (13.8%)		
30–39	85 (63.9%)	44 (58.7%)	41 (70.7%)		
40–49	29 (21.8%)	21 (28.0%)	7 (12.1%)		
Missing data	3 (2.3%)	1 (1.3%)	2 (3.4%)		
Spouses’ age				3.38	0.185
20–29	9 (6.7%)	5 (6.6%)	4 (6.9%)		
30–39	75 (56.4%)	38 (50.7%)	37 (63.8%)		
40–49	33 (24.8%)	23 (30.7%)	10 (17.2%)		
50–59	3 (2.3%)	3 (4%)	NA		
Missing data	13 (9.8%)	6 (8%)	7 (12.1%)		
Duration of residence				NA	NA
<1 year	NA	NA	2 (3.4%)		
1–10 years	NA	NA	26 (44.8%)		
10–20 years	NA	NA	27 (46.6%)		
>20 years	NA	NA	3 (5.2%)		
Educational attainment				2.87	0.412
Junior high school	1 (0.8%)	0 (0.0%)	1 (1.7%)		
Senior high school	3 (2.3%)	2 (2.7%)	1 (1.7%)		
Technical school or college	22 (16.5%)	15 (20%)	7 (12.1%)		
University or graduate school	107 (80.5%)	58 (77.3%)	49 (84.5%)		
Marital status				1.57	0.210
Married	131 (98.5%)	73 (97.3%)	58 (100%)		
Divorced	2 (1.5%)	2 (2.7%)	0 (0.0%)		
Working status				2.45	0.484
Full-time work	71 (53.4%)	43 (57.3%)	28 (48.3%)		
Part-time work	19 (14.3%)	11 (14.7%)	8 (13.8%)		
Self-employment	11 (8.3%)	4 (5.3%)	7 (12.1%)		
Student or housewife/ househusband	32 (24.1%)	17 (22.7%)	15 (25.9%)		
Family members living together				4.135	0.042
Nuclear family	122 (91.7%)	72 (96.0%)	50 (86.2%)		
Economic status ^b^				20.63	<0.001
Excellent	2 (1.5%)	2 (2.7%)	0 (0.0%)		
Good	36 (27.1%)	30 (40.0%)	6 (10.3%)		
Fair	65 (48.9%)	26 (34.7%)	39 (67.2%)		
Bad	29 (21.8%)	17 (22.7%)	12 (20.7%)		
Poor	1 (0.8%)	0 (0.0%)	1 (1.7%)		
Number of children				13.32	0.001
1	64 (48.1%)	27 (36.0%)	37 (63.8%)		
2	48 (36.1%)	30 (40.0%)	18 (31.0%)		
≥3	21 (15.8%)	18 (24.0%)	3 (5.2%)		
Youngest child’s age				9.056	0.170
0 year	22 (16.5%)	15 (20.0%)	7 (12.1%)		
1 year	32 (24.1%)	18 (24.0%)	14 (24.1%)		
2 years	12 (9%)	6 (8.0%)	6 (10.3%)		
3 years	16 (12%)	6 (8.0%)	10 (17.2%)		
4 years	14 (10.5%)	5 (6.7%)	9 (15.5%)		
5 years	17 (12.8%)	10 (13.3%)	7 (12.1%)		
6 years	20 (15%)	15 (20.0%)	5 (8.6%)		
Youngest child’s sex				0.22	0.638
Female	65 (48.9%)	38 (50.7%)	27 (46.6%)		
Male	68 (51.1%)	37 (49.3%)	31 (53.4%)		
Presence of children with chronic diseases or disabilities				1.80	0.18
Female	65 (48.9%)	38 (50.7%)	27 (46.6%)		
Presence of children with chronic diseases or disabilities				1.80	0.18
No	127 (95.5%)	70 (93.3%)	27 (98.2%)		
Yes	6 (4.5%)	5 (6.7%)	1 (1.8%)		

Note. NA = Not Applicable. ^a^ Values were calculated by *t*-test, chi-squared test, or Mann–Whitney U test; ^b^ The economic conditions in this study were based on the subjective economic status of participants.

**Table 2:** Bivariate analysis between key variables (*n* = 133).

	Total	Japanese (*n* = 75)	Chinese (*n* = 58)			Family Functioning		Family Satisfaction	
	Median (IQR)	Median (IQR)	Median (IQR)	Z	*p* ^f^	*r* ^g^	*p*	*r* ^g^	*p* ^h^
Bonding SC ^a^	22 (18–25)	22 (18–25)	22 (18–25)	0.28	0.904	−0.27	0.002	0.26	0.003
Bridging SC ^b^	18 (14–21)	17 (14–20)	18 (16–22)	2.00	0.074	0.00	0.962	−0.02	0.835
Depressive symptoms ^c^	4(1.5–7)	3 (1–6)	5 (3–7)	2.29	0.004	0.45	<0.001	−0.25	0.003
Family functioning ^d^	22 (16.5–25)	20 (15–23)	24 (18.75–26.52)	−3.23	0.001		NA	−0.68	<0.001
Family satisfaction ^e^	2 (1–2)	2 (1–2)	2 (2–2)	3.10	0.001	−0.68	<0.01		NA

Notes. Bonding SC = Bonding social capital; Bridging SC = Bridging social capital; IQR = Interquartile range. ^a^ The score of Bonding SC was calculated using the Personal Social Capital Scale-16 (items 1–8 assess bonding social capital); the higher the score, the higher the level of Bonding SC. ^b^ The score of Bridging SC was calculated using the Personal Social Capital Scale-16 (items 9–16 assess bridging social capital); the higher the score, the higher the level of Bridging SC. ^c^ Kessler Psychological Distress Scale (K6). ^d^ The General Functioning Subscale of the Family Assessment Device Scale. ^e^ The family satisfaction item of the Japanese and Chinese versions of the New Brief Job Stress Questionnaire. ^f^ Values were calculated by Mann–Whitney U test. ^g^ The correlation coefficient is the correlation coefficient with the total participants. ^h^ Values were calculated using Spearman rank correlation tests. NA = Not Applicable.

**Table 3:** Comparisons of model fit indicators between the model without path constraints and models with all-path constraints.

Models	CMIN	Change ^a^	*p* ^b^	CMIN/df	CFI	AGFI	GFI	RMSEA	AIC
Model 1 (Model without any path constraints)	0.984			0.246	1.000	0.978	0.997	0.000	52.984
Models with path constraints									
Model 2 (All paths)	9.358	8.040	0.154	1.040	0.997	0.911	0.973	0.017	51.358
Model 3 (Bonding social capital to parents’ depressive symptoms)	1.787	0.803	0.370	0.357	1.000	0.967	0.994	0.000	51.787
Model 4 (Bridging social capital to parents’ depressive symptoms)	2.065	1.081	0.298	0.413	1.000	0.963	0.994	0.000	52.065
Model 5 (Bonding social capital to family well-being)	2.748	1.878	0.171	0.550	1.000	0.950	0.992	0.000	52.748
Model 6 (Bridging social capital to family well-being)	1.044	0.022	0.883	0.209	1.000	0.981	0.997	0.000	51.044
Model 7 (Parents’ depressive symptoms to family well-being)	5.789	4.391	0.036	1.158	0.994	0.898	0.983	0.035	55.789

Notes. AGFI = adjusted goodness-of-fit index; AIC = Akaike’s information criterion; CMIN = Chi-squared statistics; CFI = comparative fit index; GFI = goodness-of-fit index; RMSEA = root mean square error of approximation. ^a^ Chi-squared changes compared to the chi-square of the model without any path constraints. ^b^ *p*-value was calculated to indicate the significance of chi-squared changes.

## Data Availability

The data presented in this study are available on request from the corresponding author due to no approval from the Ethics Committee of Kobe University to share the collected data publicly.
